# Identifying hub genes of calcific aortic valve disease and revealing the immune infiltration landscape based on multiple WGCNA and single-cell sequence analysis

**DOI:** 10.3389/fimmu.2022.1035285

**Published:** 2022-11-04

**Authors:** Kan Wang, Qiang Zheng, Xing Liu, BingChuan Geng, NianGuo Dong, JiaWei Shi

**Affiliations:** Department of Cardiovascular Surgery, Union Hospital, Tongji Medical College, Huazhong University of Science and Technology, Wuhan, Hubei, China

**Keywords:** calcific aortic valve diseases (CAVD), DEGs, weighted gene co-expression network analysis (WGCNA), single cell sequence (scRNA-seq), immune infiltration, bioinformatics

## Abstract

**Background:**

Calcific aortic valve disease (CAVD) is a progressive fibrocalcific disease that can be treated only through valve replacement. This study aimed to determine the role of hub genes and immune cell infiltration in CAVD progression.

**Methods:**

In this study, bioinformatics analysis was used to identify hub genes involved in CAVD. The datasets were downloaded from the Gene Expression Omnibus (GEO) database. Gene expression differences were evaluated *via* pathway and Gene Ontology analyses. Weighted gene co-expression network analysis (WGCNA) and differentially expressed genes were used to screen hub genes. The CIBERSORT algorithm was used to compare immune infiltration into the calcified aortic valve based on the hub genes between high- and low-expression groups. We also performed single-cell RNA sequencing based on six different human aortic valve leaflets. The expression of hub genes was identified in human and mouse samples through quantitative real-time polymerase chain reaction (qPCR), immunohistochemistry, immunofluorescence, and ELISA, and clinical features of the patients were investigated.

**Results:**

In total, 454 differentially expressed genes were obtained from the GEO database. WGCNA was used to find 12 co-expression modules in the Array Express database, of which one hub module (brown module) was most correlated with CAVD. Two hub genes were identified after combining the differentially expressed genes *S100A8* and *S100A9*. Regarding these genes, the immune infiltration profiles varied between high- and low-expression groups. Compared with that in the low hub gene expression group, the high hub gene expression group had a higher proportion of activated NK cells (*p* < 0.01) and M1 macrophages (*p* < 0.05). The expression of *S100A8* and *S100A9* was consistent with single-gene RNA sequencing results, confirming that the expression levels of these two hub genes are significantly upregulated in patients with CAVD (*p* < 0.01). Furthermore, these results were verified using mouse and human samples by performing immunofluorescence, immunohistochemistry, qPCR, and ELISA analyses. Finally, the localization of S100A8 and S100A9 in monocytes and macrophages was confirmed *via* immunofluorescence using human aortic valves.

**Conclusion:**

These results demonstrate that *S100A8* and *S100A9* are two hub genes involved in CAVD, which might play an important role in its development through immune-related signaling pathways.

## Introduction

Calcific aortic valve disease (CAVD) is one of the most common heart valve diseases in the world (1, 2). According to statistics, its prevalence is 2% in patients >60 years of age, and with the aging population, its prevalence is increasing gradually (3). CAVD is increasingly recognized as a serious public health concern with a 2-year mortality rate of 50% in severely ill patients (4, 5).

Previous studies have shown that the mechanisms of CAVD depend on complex pathophysiological processes, including an initial endothelial injury, immune cell infiltration, and valvular interstitial cell (VIC) myofibroblast differentiation. Calcification of the aortic valve occurs following the trans-differentiation of the VICs, *via* a myofibroblast stage, into osteoblast-like cells (6). Disease progression is inevitable once mild valve stenosis occurs. However, the only curative therapeutic modality for CAVD is aortic valve replacement owing to the lack of valid pharmacotherapeutics (7). At the same time, owing to the limiting effects of advanced age and comorbidities on overall survival, the prognosis of patients with severe CAVD is poor (8). Therefore, it is necessary to understand disease pathogenesis and the molecular changes that occur.

Recently, with the advent of next-generation sequencing technology, genome sequencing has increased dramatically. However, acquiring large sample size data is not always practical because of tissue and time constraints, particularly in clinical settings. Therefore, pooled analysis is required for different databases.

Here, we first extracted five gene datasets from two publicly available databases (Gene Expression Omnibus and Gene Card). Then, weighted gene co-expression network analysis (WGCNA) and differentially expressed gene (DEG) screening were used to identify differentially co-expressed genes. Single-cell RNA sequencing (scRNA-seq) data from our center further revealed the expression patterns of hub genes in CAVD cell clusters. We then assessed the association between immune cell infiltration and hub genes. Finally, we successfully validated the expression of hub genes in both mouse and human aortic valves.

## Materials and methods

### Human samples and scRNA-seq analysis

Calcified aortic valves were harvested from patients with CAVD who had undergone aortic valve replacement surgery. Healthy aortic valves were obtained from patients undergoing aortic dissection repair that required aortic valve replacement. All human aortic valve samples were obtained from the Wuhan Union Hospital. The preparation of single-cell suspensions and the scRNA-seq procedure for aortic valves have been described previously in detail (9). Low-quality cells were removed according to the percentage of mitochondrial gene expression. The data were then integrated and standardized for downstream analyses. The Seurat pipeline was used for data reprocessing and to classify cell groups. SingleR was used to identify the cell type, and Monocle was used to analyze the cell differentiation trajectory. Fifty-five blood serum samples were used for ELISA. Eighteen aortic valves were used for qPCR. Six aortic valves were used for immunohistochemistry. Detailed patient information is presented in **Table 1**. The study protocol was approved by the Ethics Committee of the Tongji Medical College, Huazhong University of Science and Technology, and a waiver of informed consent was remitted.

**Table 1:** The detailed information on each patients.

Variables	Normal	CAVD	Total n=55 (%)
**N**	20	35	55
**Sex**	/	/	/
Male	8 (40.0)	27 (77.1)	35 (63.6)
Female	12 (60.0)	8 (22.9)	20 (36.4)
**Age (year)**	55.25 (4.10)	59.86 (9.71)	58.18 (8.38)
**BMI(kg/m2)**	22.76 (3.29)	24.95 (3.55)	24.15 (3.58)
**NYHA (%)**	/	/	/
1	10 (50.0)	3 (8.6)	13 (23.6)
2	10 (50.0)	9 (25.7)	19 (34.5)
3	0	16 (45.7)	16 (29.1)
4	0	7 (20.0)	7 (12.7)
**BMI(kg/m2)**	22.76 (3.29)	24.95 (3.55)	24.15 (3.58)
**EF(%)**	62.70 (4.08)	58.46 (9.29)	60.00 (8.03)
**Echocardiography**	/	/	/
PAJV (cm/s)	1.22 (0.22)	3.85 (1.06)	2.89 (1.54)
IVST(cm)	0.98 (0.15)	1.22 (0.24)	1.13 (0.24)
**Lab experiment**	/	/	/
BNP(pg/ml)	59.27 (79.67)	352.19 (577.10)	245.68 (481.82)
Cr(μmol/L)	69.60 (14.60)	84.00 (25.34)	78.76 (22.99)
Ca^2+^(mmol/L)	2.20 (0.11)	2.22 (0.13)	2.21 (0.12)
P(mmol/L)	1.09 (0.32)	1.17 (0.34)	1.14 (0.33)
**History**	/	/	/
Hypertension	6	18	24
Diabetes	0	6	6
Smoking	5	18	22

PAJV, Peak aortic jet velocity; IVST, interventricular septum thickness; CAD, coronary heart disease.

### Aortic valve calcification animal experiment

Male ApoE−/− C57BL/6 mice, aged 8–10 weeks, were purchased from the Experimental Animal Center of Huazhong University of Science and Technology (Wuhan, China). Animal experimental protocols were approved by the Animal Care and Utilization Committee of Tongji Medical College, Huazhong University of Science and Technology. ApoE−/− mice were fed in a suitable environment and divided into two groups as follows: mice fed a normal diet (n = 6) and mice fed a western diet (WD; cat. #88137; Harlan Laboratories, Ferndale, WA, USA; n = 6) to induce aortic valve calcification. After 24 weeks, the final transthoracic echocardiography and hemodynamic assessments were performed, the mice were euthanized, and tissues were collected.

### Data collection and preprocessing

Gene expression profile data (GSE51472, GSE153555, GSE83453, and GSE12644) were downloaded from the GEO database of the National Center for Biotechnology Information (NCBI; https://www.ncbi.nlm.nih.gov/geo/). GSE51472, GSE83453, and GSE12644 comprise microarray data, whereas GSE153555 comprises RNA-Seq data. The GSE51472 dataset is based on the GPL570 platform [HG-U133_Plus_2] Affymetrix Human Genome U133 Plus 2.0 Array, which was submitted by Rysa et al. (10). The GSE153555 dataset is based on the GPL16791 Illumina HiSeq 2500 dataset (*Homo sapiens*) *(* 11). The GSE83453 dataset is based on the GPL10558 Illumina HumanHT-12 V4.0 Expression Beadchip (12). The GSE12644 dataset is based on GPL570, which was submitted by Bossé et al. (13). The GSE51472, GSE153555, GSE83453, and GSE12644 datasets contain 91 samples, including 59 CAVD samples and 32 normal aortic valve tissue samples (12). The CAVD datasets from different GEO datasets are summarized (**Supplementary File 7**). We downloaded 1,651 CAVD-related genes based on the keyword “calcified aortic valve disease” from GeneCards (https://www.genecards.org/).

### Data processing and DEG identification

The affy package (http://www.bioconductor.org/packages/release/bioc/html/affy.html) (14) in R (version 4.2.1, https://www.r-project.org/) was used to preprocess and normalize the raw data. The matrix data of GSE51472 and GSE12644 were merged and normalized using the sva package in R software. The DEGs between CAVD samples and normal aortic valve samples were identified using the limma package (http://www.bioconductor.org/packages/release/bioc/html/limma.html) (15) in R. Genes with *P*-values <0.05 and a |logFC| >1 were considered DEGs. The overlapping DEGs between the datasets are shown in a Venn diagram. We compared the list of DEGs with that in the original studies. After adjusting for the screening conditions, the results were not different from those of our analysis.

### Functional enrichment analysis

We used the DAVID website to analyze DEGs based on KEGG pathway enrichment and Gene Ontology (GO) functional analysis (https://david‐d.ncifcrf.gov/) (16). Then, The GO and KEGG output was visualized using R 4.2.1 software (ggplot2 package) (17). A Benjamini–Hochberg-adjusted *P*-value (Benjamini and Hochberg, 1995) <0.05 was set as the cutoff criterion.

### WGCNA

We used the “WGCNA” package in R software to create a co-expression network for DEGs (18). Clinical traits were classified into two types, normal and CAVD, to identify module–trait relationships. Sample clustering was performed to match the sample characteristics. We performed this analysis using the GSE12644 dataset. Fifteen samples were included in this analysis. An appropriate soft-thresholding power was chosen with an ideal soft‐thresholding power of 16. For each expression profile, module membership and gene significance were defined as the correlation values for each trait and module eigengene.

### Immune cell infiltration estimation

The CIBERSORT deconvolution algorithm was used to quantify immune cell infiltration using transcriptome data. This was achieved using the CIBERSORT algorithm based on deconvolution using the “CIBERSORT” R package (CIBERSORT R script v1.03; http://cibersort.stanford.edu/). The differences between the S100A8/S100A9 high- and low-expression groups were compared using the Wilcoxon test. Results were visualized using the vioplot package. Finally, the correlation between the infiltration rate of each type of immune cell was determined using the corrplot package.

### RT-PCR assay

Total RNA from aortic valves was extracted using TRIzol Reagent (Life Technologies, Los Angeles, CA, USA). cDNA synthesis was performed using a cDNA synthesis kit (Vazyme; R101-01). Primers used are listed in **Table 1**. According to the kit instructions, the primers (Shanghai Bioengineering), cDNA, ChamQ Universal SYBR qPCR Master Mix, and non-RNase ddH_2_O were thoroughly mixed and then added to a 96-well PCR plate (Bio-Rad, Los Angeles, CA, USA), which was then placed into a real-time PCR detection system (Bio-Rad). Results were normalized to *GAPDH* levels using the ΔΔCt method (**Table 2**).

**Table 2:** Primer sequences used for quantitative real-time PCR (RT-PCR).

Primer	Forward sequences (5′–3′)	Reverse sequences (5′–3′)
**S100A8**	CCTGAAGGTTCTGTTTTTCAGGTG	CACGCCCATCTTTATCACCA
**S100A9**	TGGAGGACCTGGACACAAATG	CACCCTCGTGCATCTTCTCG
**GAPDH**	TACCACATCCAAGGAACAGCA	TGGAATTACCGCGGCTGCTGGCA

### Immunohistochemistry

Briefly, valve tissues were collected and fixed in 4% paraformaldehyde. Tissues were then embedded in paraffin. Sections (5 μm) were prepared for immunohistochemistry. Staining was performed using antibodies against S100A8 (A12018; ABclonal, China) and S100A9 (A9842; ABclonal). The specimens were observed under a confocal laser scanning microscope (Olympus, Tokyo, Japan), and the area of staining was quantified using ImageJ software.

### ELISA

The human S100A8/S100A9 ELISA kit (F10998-B, F10191-B; Fankew) was used to measure the levels of these proteins in plasma samples. All steps were performed according to the manufacturer’s instructions (Shanghai Frankel Industrial Co., Ltd.; Fankew). All plasma protein levels are expressed as pg/mL.

### Effect of immune checkpoint-, m6A-, ferroptosis-, and cuprotosis-related gene expression in CAVD

We identified immune checkpoint-, ferroptosis-, cuprotosis-, and m6A-related genes based on previous studies. First, we divided hub genes into high- and low-expression groups. We then performed a multigene Spearman correlation analysis of the immune checkpoint, ferroptosis, and m6A methylation to describe the correlation among immune checkpoints, ferroptosis, cuprotosis, and m6A genes. Statistical significance was set at *P* < 0.05. In addition, box plots and heatmaps were obtained using the boxplot, ggord, and pheatmap packages in R. All of these analysis methods were performed using R version 4.2.1.

## Results

**Figure 1:**
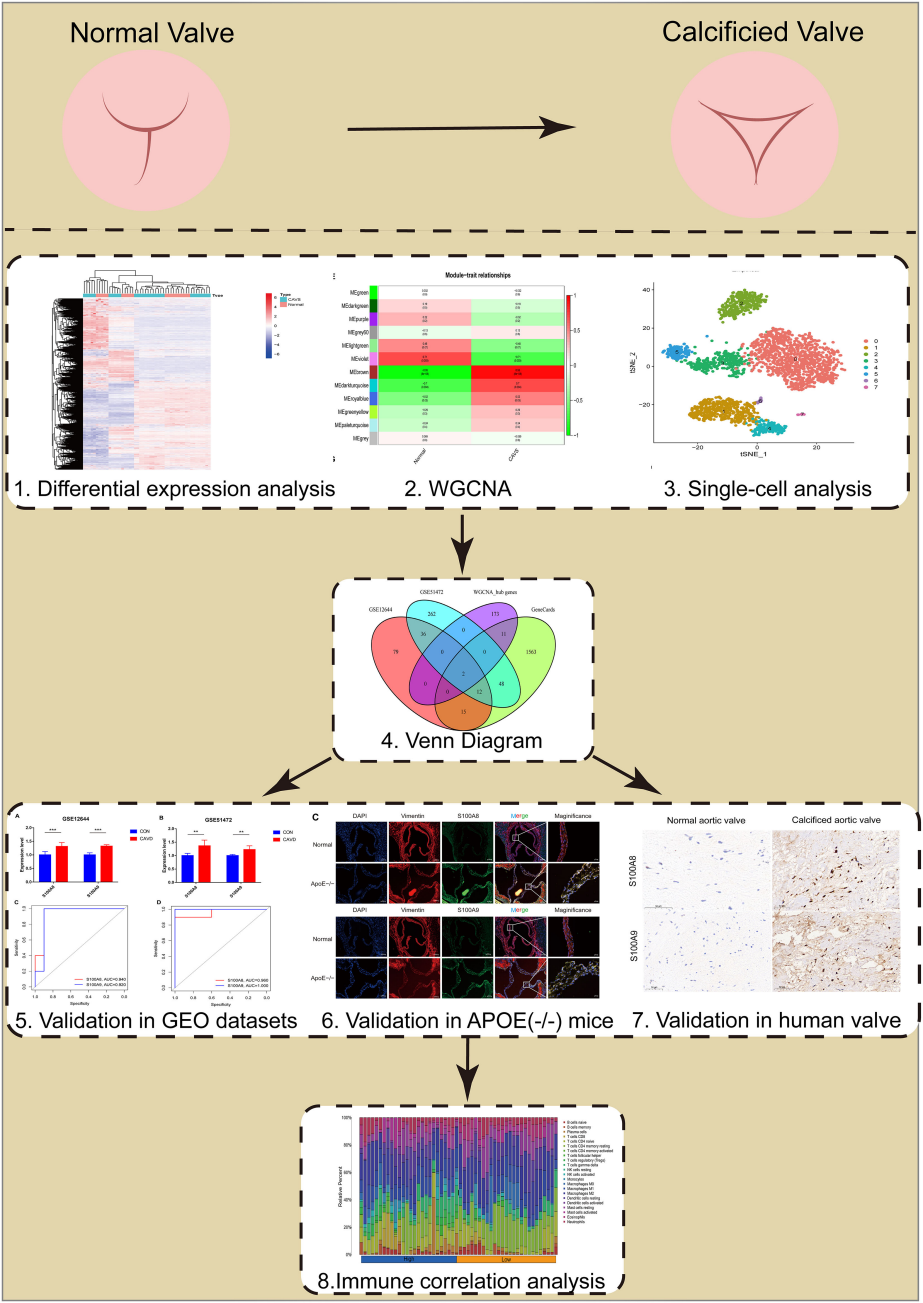
Flowchart of our study.

The flowchart of our study is shown in **Figure 1**.

### Screening of DEGs

**Figure 2:**
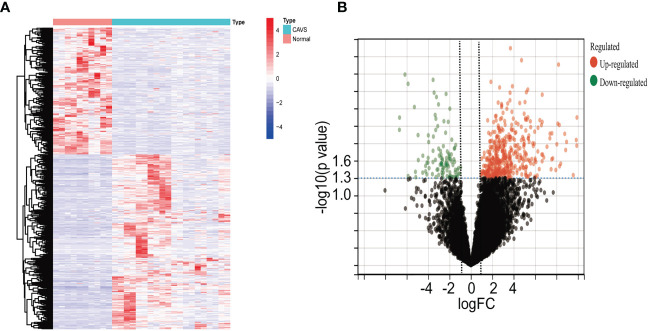
Differential gene analysis of GSE51472 and GSE153555 datasets. **(A)** Heatmap of the combined GEO database. Green represents downregulated genes, and red represents upregulated genes. **(B)** Volcano plot of the combined GEO database. Left green plots represent genes that are expressed at low levels in calcific aortic valve disease (CAVD) tissue, and right red plots represent genes that are expressed at high levels in CAVD tissue.

Four datasets were downloaded from the GEO database. After data collection, the microarray data were normalized. When identifying DEGs, a threshold fold change of 1 and a *P*-value of 0.05 were used. The limma package was used to analyze the DEGs in GSE51472 and GSE12644 datasets. GEO data were merged, and the merged code is shown in **Supplementary File 4**. Based on the aforementioned criteria, 15 normal aortic valve samples and 20 calcific aortic valve samples from the GEO dataset were analyzed. In the combined GEO database, 259 DEGs were obtained after removing the batch differences and performing data normalization, including 191 upregulated and 68 downregulated DEGs (**Figures 2A, B**; **Supplementary File 1**). Meanwhile, 2,108 DEGs were identified in GSE153555; expression levels of 1,140 genes were upregulated, and those of 968 genes were downregulated (**Supplementary File 5**).

### Functional enrichment analysis

**Figure 3:**
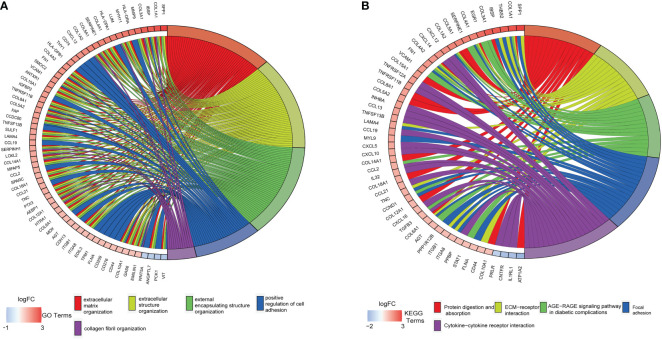
Enrichment analysis of GSE51472 and GSE153555 datasets. **(A)** Gene Ontology (GO) enrichment. **(B)** Kyoto Encyclopedia of Genes and Genomes enrichment (KEGG).

The DAVID tool was used to examine the potential functions of these DEGs by determining GO and KEGG pathway enrichment. The GO analysis results consisted of biological processes, cellular components, and molecular functions. As shown in **Figure 3A**, upregulated DEGs were primarily involved in “extracellular matrix organization”, “extracellular structure organization”, “external encapsulating structure organization”, “positive regulation of cell adhesion”, and “collagen fibril organization”. The significantly enriched KEGG pathways related to upregulated DEGs in CAVD were associated with “protein digestion and absorption”, “ECM–receptor interaction”, “AGE-RAGE signaling pathway in diabetic complications”, and “focal adhesion” and “cytokine–cytokine receptor interaction” terms (**Figure 3B**).

### WGCNA and identification of hub genes

**Figure 4:**
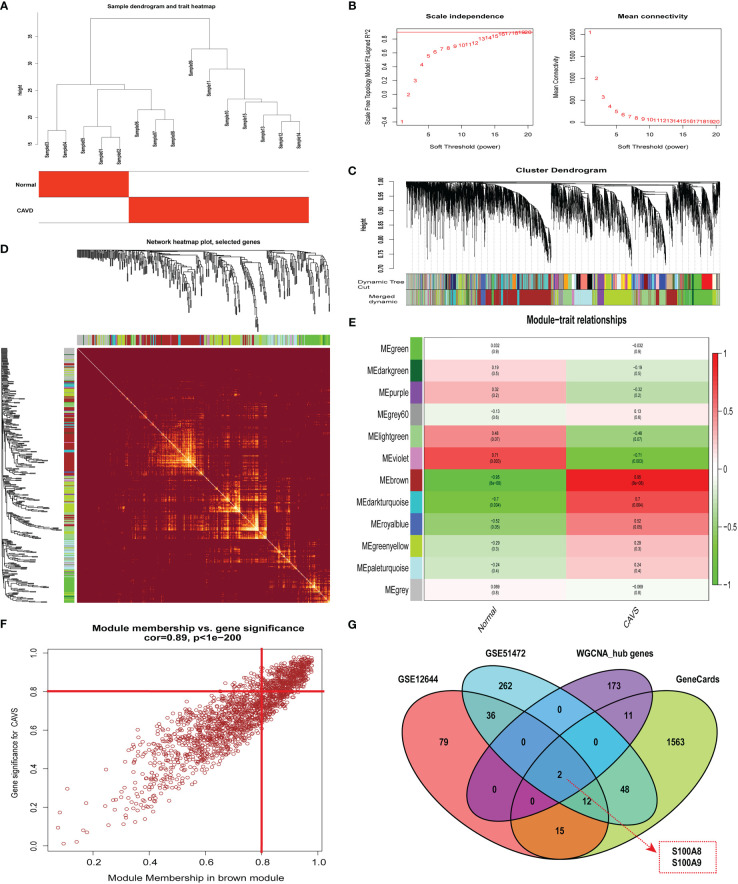
Weighted co-expression network analysis (WGCNA) of GSE12644 dataset and Venn diagram to obtain the key genes *S100A8* and *S100A9*. **(A)** Sample clustering of the GSE12644 dataset. The sample was clustered into two significantly different clusters. **(B)** Selection of optimal thresholds. The threshold was 16. **(C)** By aggregating genes with strong correlations in the same module, different modules were obtained and are displayed in different colors. **(D)** A network heatmap plot was created through WGCNA showing overall module-related gene branches in hierarchical clustering dendrograms. **(E)** Correlation analysis between modules and calcific aortic valve disease (CAVD). **(F)** The brown module was significantly positively correlated with CAVD (COR = 0.95, *P* < 0.001). The genes in the brown module are labeled as the WGCNA-hub genes. **(G)** Intersection of GeneCards, WGCNA, and differential expression data, displayed in the Venn diagram. The two most significant genes, *S100A8* and *S100A9*, were obtained.

By clustering the 15 samples in GSE153555, we found that the five samples on the left and the 10 samples on the right were quite different and formed two clusters (**Figure 4A**). As a result of our calculations, we chose 34 as the cutoff value and included the remaining 14 samples in the subsequent analysis. The soft threshold power value was set to 16 for subsequent analyses (**Figure 4B**). In addition, a dendrogram and heatmap were used to quantify module similarity based on the correlation (**Figures 4C** and **D**). Associations between clinical traits and modules were identified based on these correlations. Brown modules were positively correlated with CAVD (correlation = 0.95, *P* < 0.001, **Figure 4E**), suggesting that it plays an important role in calcified and normal aortic valves, which might be related to the occurrence and development of CAVD. Defined based on a combined gene significance >0.8 and module membership >0.8 among genes in the brown module, 186 total genes that were highly connected in the brown module were identified as candidate hub genes (**Figure 4F**). Finally, the intersection of the gene card, WGCNA, and differential expression data are displayed in the Venn diagram. The two most significant genes, *S100A8* and *S100A9*, were obtained (**Figure 4G**). WGCNA-associated genes are listed in **Supplementary File 6**.

### Single-cell sequencing data analysis

**Figure 5:**
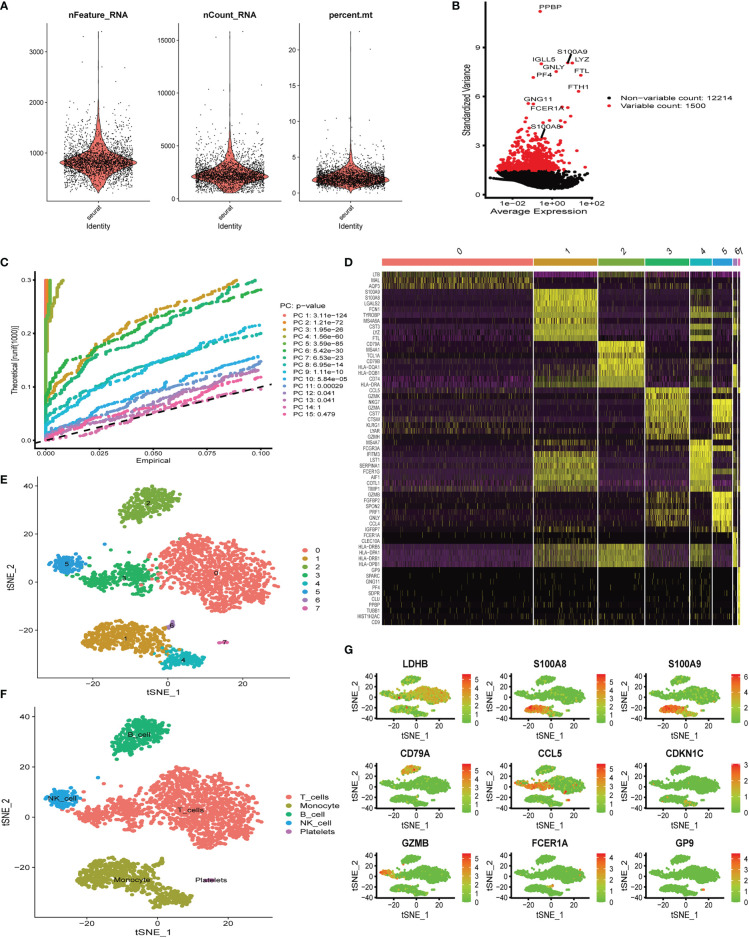
Single-cell quality control and dimension reduction clustering at our center. **(A)** The percentage of mitochondrial genes and erythrocyte genes was limited to ensure the reliability of cell samples; **(B)** 1,500 highly variable genes are shown in red, with the top 10 highlighted. **(C)** Principal component analysis (PCA) of the single-cell expression profiles. **(D)** Heatmap of expression patterns of cluster-enriched genes. **(E, F)** Dimensionality reduction and cluster analysis. The cells in the valve could be divided into eight clusters, which could be approximately summarized as T cells, monocytes, B cells, NK cells, and platelets. **(G)** Expression of marker genes in the main cell clusters based on the tSNE map.

Six aortic valve tissue samples obtained from our center were digested to prepare single-cell suspensions. In addition, because of the tight link between hub genes (*S100A8* and *S100A9*) and immune function (19–21), we only extracted immune-related cells for single-cell analysis in the following studies. Quality control analysis of a single-cell dataset was performed. As shown in **Figure 5A**, three indicators—RNA count, gene count, and percentage of mitochondrial genes (nCount_RNA, nFeature_RNA, and percent.mt)—were chosen to demonstrate the reliability of the data. We then selected 1,500 highly variable genes and tagged the top 10 genes. In **Figure 5B**, all hypervariable genes are marked in red. To further confirm that the cells analyzed belonged to different cell clusters, we performed normalization and principal component analysis (PCA) with scRNA-seq samples (**Figure 5C**). In **Figures 5D, E**, eight clusters were found among the cells, and we generated a heatmap of the marker genes for each cluster. Then, cell types were classified based on the expression of key marker genes (**Figure 5D**), and finally, five cell types were determined, T cells, monocytes, B cells, NK cells, and platelets (**Figure 5F**). Genes can be candidate marker genes of each cluster and are displayed in tSNE diagrams (**Figure 5G**).

### Pseudotime analysis

**Figure 6:**
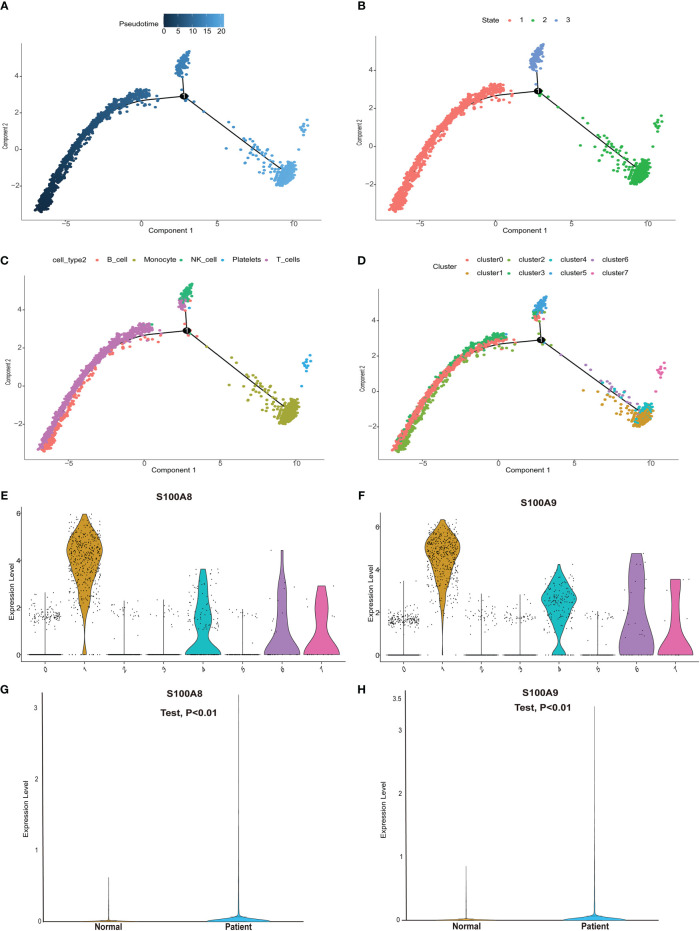
Pseudotime analysis and expression level of hub genes in the single-cell dataset. **(A)** Differences in the time sequence of cell differentiation. Darker blue indicates earlier differentiation, and lighter blue indicates later differentiation. This provided a reference for subsequent analysis. **(B)** Three differentiation states of calcified valve diseases. The differentiation in state 2 occurred at the latest timepoint. **(C)** Differences in differentiation among five different cell types. **(D)** All cells were clustered into eight clusters. **(E, F)** Analysis of hub gene (*S100A8*/*S100A9*) expression based on our single-cell sequencing dataset from our center. *S100A8*/*S100A9* expression was upregulated in cluster groups (1, 4, 6, 7), which suggested that hub gene expression is higher in the latest stage of calcific aortic valve disease (CAVD). **(G, H)** *S100A8*/*S100A9* expression analysis in the single-cell dataset. *S100A8*/*S100A9* expression was upregulated in CAVD.

We next performed pseudotime analysis to predict cell trajectories with respect to the immune cells (**Figures 6A–D**). One root and two branches were identified in single-cell analysis. Notably, cells in clusters 0, 2, and 3 were mainly located in the root, whereas those in cluster 5 were mostly located in the middle stage, and most cells in clusters 1, 4, 6, and 7 were located at the end stage (**Figure 6D**). We performed single-cell sequencing analysis to explore the expression of the modeling genes in different cell clusters. As shown in **Figures 6E, F**, S100A8 and S100A9 were mainly expressed in clusters 1, 4, 6, and 7. Single cells and clusters of cells from normal (aortic valve) tissues and clusters of calcified aortic valve cells were used. The expression of hub genes (S100A8 and S100A9) was upregulated in the single-cell sequence data (*P* < 0.01, **Figures 6G, H**).

### Validation of hub genes in the GEO database

**Figure 7:**
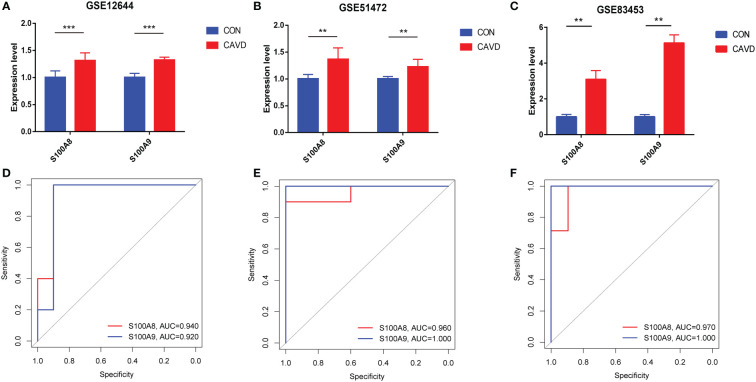
Diagnostic performance of S100A8/S100A9 expression levels for calcific aortic valve disease (CAVD) patients in the datasets. **(A–C)** The expression levels of *S100A8*/*S100A9* in CAVD were based on the GSE databases (GSE153555, GSE51472, and GSE83453). **(D–F)** Diagnostic value of *S100A8* and *S100A9* in GSE databases (GSE153555, GSE51472, and GSE83453). Receiver operating characteristic (ROC) curve of normal and CAVD valve tissues. ^**^ *P* < 0.01, ^***^ *P* < 0.001 when two groups were compared as indicated or compared with the corresponding control.

After normalizing the raw data, we validated the expression of *S100A8*/*S100A9* in the GSE51472, GSE12644, and GSE83453 datasets. As shown in **Figures 7A–C**, the expression of *S100A8* and *S100A9* was higher in patients with CAVD than in healthy controls (*P* < 0.05). The corresponding areas under the curve (AUCs) for these two hub genes were also obtained, including those of *S100A8* (AUC = 0.940, AUC = 0.960, AUC = 0.970) and *S100A9* (AUC = 0.920, AUC = 1.000, AUC = 1.00) in three databases (**Figures 7D–F**). Notably, these data demonstrate that these two genes (*S100A8* and *S100A9*) might also be accurate biomarkers for patients with CAVD.

### Hub genes are associated with immune checkpoints, but not m6A, cuproptosis, or ferroptosis, in CAVD

**Figure 8:**
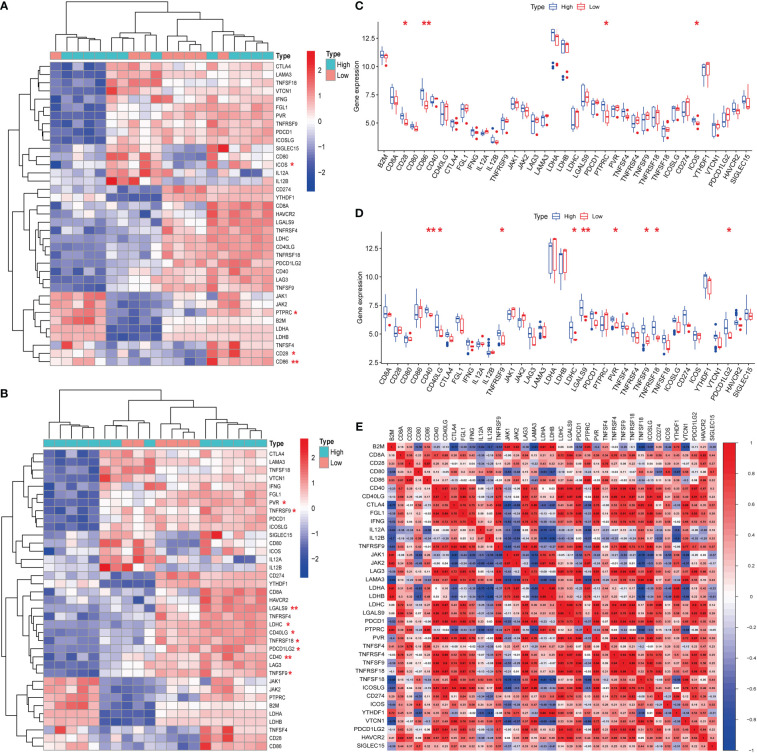
Expression of immune checkpoint-related mRNAs in calcific aortic valve disease (CAVD) patients. **(A, B)** Comparison of expression levels of 38 immune checkpoint-related RNAs. Immune checkpoint-related RNA expression between *S100A8*/*S100A9* high- and low-expression groups of patients. Blue represents high expression, whereas red represents low expression. **(C, D)** Visualization of differentially expressed immune checkpoint-related genes in CAVD. *S100A8*/*S100A9* high expression in CAVD patients is marked with blue, and S100A8/S100A9 low expression is marked with red. **(E)** Spearman correlation analysis of 38 immune checkpoint-related RNAs in CAVD. A higher number in the circle indicates a stronger correlation. The change in color on the right represents a positive or negative correlation. ^*^ *P* < 0.05, ^**^ *P* < 0.01, ^***^ *P* < 0.001 when two groups were compared as indicated or compared with the corresponding control.

Hub genes (*S100A8* and *S100A9*) were obtained from a previous analysis. Based on the expression of these genes, we divided these samples into high- and low-expression groups. From the immune checkpoint-, m6A-, proptosis-, and ferroptosis-associated genes that were collected, changes were observed in gene expression between patients in the high- and low-expression groups. Notably, we observed that the immune checkpoint-related genes *ICOS*, *PTPRC*, *CD28*, and *CD86* were closely associated with the expression of *S100A8* (**Figures 8A, C**). In addition, the immune checkpoint-related genes *PVR*, *TNFRSF9*, *LGALS9*, *LDHC*, *CD40LG*, *TNFRSF18*, *PDCD1LG2*, *CD40*, and *TNFSF9* were closely associated with the expression of *S100A9* (**Figures 8B, D**). The strongest associations between *LAG3* and *CTLA4* and between *CTLA4* and *PDCD1* were found after analyzing immune checkpoint-related genes (**Figure 8E**). In contrast, the hub genes for which there was a change in expression showed little association with ferroptosis-, m6A-, and cuproptosis-related genes (**Figures 2**, **3**; **Supplementary Figure 1**).

### Immune cell infiltration results

**Figure 9:**
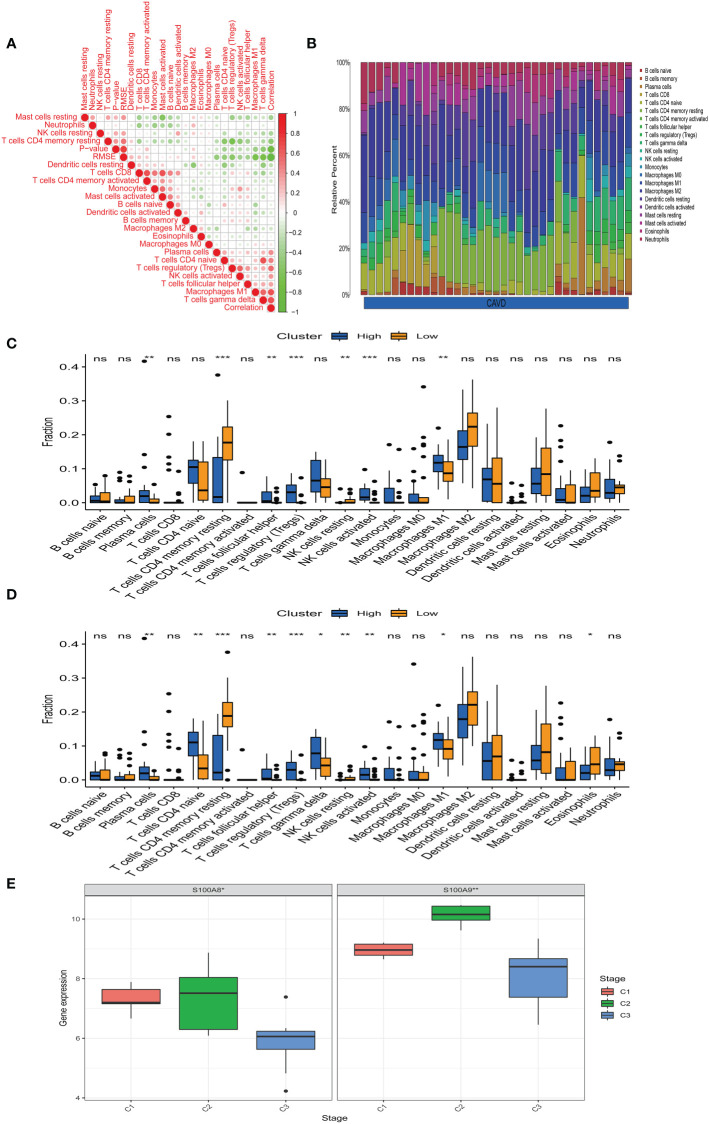
Immune infiltration analysis. **(A)** Correlation analysis of different immune cells. Darker green indicates a stronger negative correlation, and darker red shows the strongest positive correlation. **(B)** The immune landscape of calcific aortic valve disease (CAVD) revealed that the proportion of macrophages in the CAVD immune microenvironment was highest. **(C)** According to the expression of S100A8, patients were divided into a high- and low-expression groups. The infiltration levels of some immune cells (plasma cells, resting CD4 memory T cells, etc.) were different between the two groups. **(D)** According to the expression of S100A9, patients were divided into high- and low-expression groups. The infiltration level of some immune cells (plasma cells, CD4 native T cells, etc.) was different between the two groups. **(E)** Expression of S100A8 and S100A9 in immune subtypes. S100A8 and S100A9 tended to be highly expressed in the C2 immune subtype, and the difference was statistically significant (*P* < 0.05). ^*^ *P* < 0.05, ^**^ *P* < 0.01, ^***^ *P* < 0.001 when two groups were compared as indicated or compared with the corresponding control. Ns indicates non-significant.

Bioinformatic analysis confirmed that hub genes are associated with immune function. We uploaded the GSE51472 and GSE153555 datasets to CIBERSORT to analyze immune cell infiltration. A correlation analysis for these immune cells was performed (**Figure 9A**), with darker green indicating a stronger negative correlation and darker red indicating the strongest positive correlation. We found that there was a strong correlation between CD8 T cells and mast cell activation and a strong correlation between CD4 native T cells and gamma delta T cells in CAVD. Mast cell-activated cells and resting mast cells were strongly negatively correlated, whereas resting memory Tregs and resting CD4 memory T cells showed the strongest negative correlation. **Figure 9B** shows the composition of immune cells in each aortic valve. Macrophages were the dominant immune cells in calcified aortic valves. The infiltration level of M0 macrophages was different between the two groups (**Figure 9C**). After dividing the CAVD patients into high- and low-score groups based on median gene expression, we found that a high expression of *S100A8* and S100A9 was correlated with high levels of M1 macrophage, Treg, plasma cell, and activated NK cell infiltration, whereas a low expression of *S100A8* and *S100A9* was associated with higher infiltration levels of resting CD4 memory T cells (*P* < 0.05, **Figure 9D**). According to the infiltration of 22 types of immune cells, CAVD patients were clustered into three different immune subtypes (C1, C2, and C3; **Figure 9E**). Next, we explored the expression of the hub genes in these three immune subtypes. *S100A8* and *S1100A9* were highly expressed in immune subtype C2 (*P* < 0.05, **Figure 9E**).

### GSEA

**Figure 10:**
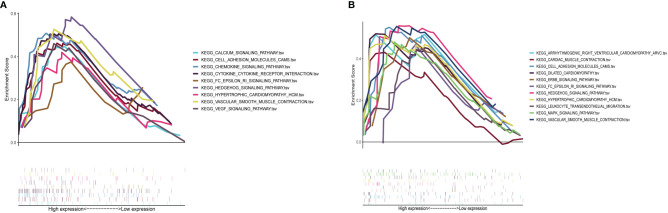
GSEA plot showing that a high expression of S100A8 and S100A9 is positively correlated with these significant signaling pathways. **(A)** In the group with high S100A8 expression in calcific aortic valve disease (CAVD), the enriched pathways included calcium signaling, cell adhesion molecules, chemokine signaling, cytokine–cytokine receptor interaction, FC epsilon RI signaling, Hedgehog signaling, hypertrophic cardiomyopathy, vascular smooth muscle contraction, and VEGF signaling. **(B)** In the group with a high expression of S100A9 in CAVD, the enriched pathways included arrhythmogenic right ventricular cardiomyopathy, cardiac muscle contraction, cell adhesion molecules, dilated cardiomyopathy, ERBB signaling, FC epsilon RI signaling, Hedgehog signaling, hypertrophic cardiomyopathy, leukocyte transendothelial migration, MAPK signaling, and vascular smooth muscle contraction.

Results of GSEA indicated that genes associated with a high S100A8 expression in the high-risk group were mainly enriched in calcium signaling, cell adhesion molecules, chemokine signaling, cytokine receptor interactions, FC epsilon RI signaling, Hedgehog signaling, hypertrophic cardiomyopathy, vascular smooth muscle contraction, and VEGF signaling (**Figure 10A**); in the group with a high *S100A9* expression in CAVD, the enriched pathways included arrhythmogenic right ventricular cardiomyopathy, cardiac muscle contraction, cell adhesion molecules, dilated cardiomyopathy, ERBB signaling, FC epsilon RI signaling, Hedgehog signaling, hypertrophic cardiomyopathy, leukocyte transendothelial migration, MAPK signaling, and vascular smooth muscle contraction (**Figure 10B**).

### Validation of S100A8 and S100A9 expression in APOE−/− mouse and human samples

**Figure 11:**
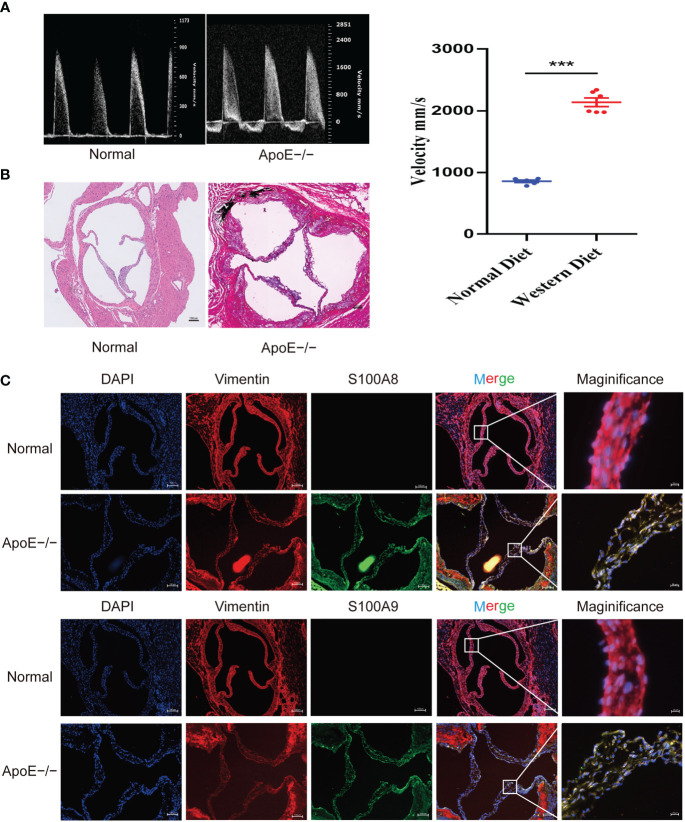
Validation of S100A8 and S100A9 expression in ApoE−/− mice. **(A)** Echocardiography of hearts from APOE−/− mice in each group. Quantification of flow-velocity maximum of the aortic valve, mm/s. Blue represents a normal diet, and red represents a western diet administered to APOE−/− mice. **(B)** Von Kossa staining of the mouse valves. The areas of valve calcification are stained black. **(C)** Immunofluorescence showed increased S100A8 and S100A9 expression levels in aortic valve tissues from ApoE−/− mice fed a western diet for 4 months. ***P< 0.001 when two groups were compared as indicated or compared to the corresponding control.

**Figure 12:**
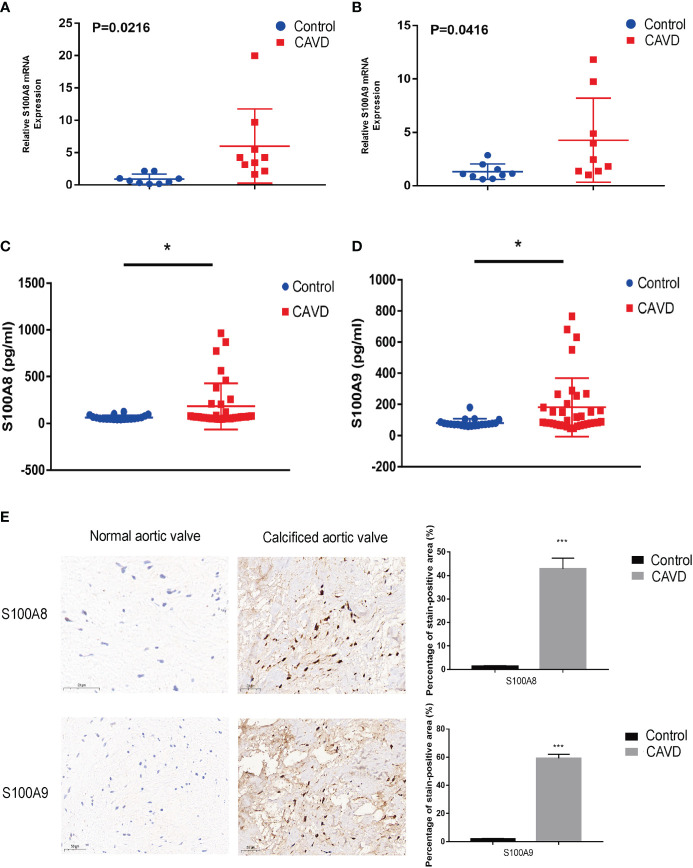
Validation of S100A8 and S100A9 expression in human samples. RT-PCR analysis of samples from nine patients with calcific aortic valve disease (CAVD) and nine normal patients. The expression of S100A8 **(A)** and S100A9 **(B)** was significantly higher in calcified aortic valves than in normal aortic valves (*P <* 0.05). Expression of S100A8 **(C)** and S100A9 **(D)** in human serum (n = 55), as determined by ELISA. **(E)** Immunohistochemical analysis and quantitative data confirmed S100A8 and S100A9 expression in calcified aortic valves. *P< 0.05, ***P< 0.001 when two groups were compared as indicated or compared to the corresponding control. Ns indicates non-significant.

To confirm these results, we established a calcified aortic valve disease mouse model using APOE−/− mice by feeding them a WD for 4 months. We found a significant difference between the measured peak flow velocity across the aortic valve between the group fed a normal diet and APOE−/− mice fed a WD for 4 months to induce CAVD (*P* < 0.05, **Figure 11A**). Meanwhile, WD-induced calcium deposition was significantly increased in the aortic tissue compared with that in the normal diet group, as assessed by silver nitrate (von Kossa) staining, wherein black/brown areas in the elastic lamina of the tunica media showed strong positive staining indicative of calcification (**Figure 11B**). The aortic valves of mice were then collected. Immunofluorescence showed that WD administration to APOE−/− mice significantly increased S100A8 and S100A9 expression (**Figure 11C**). We also examined the expression of these two markers in human samples collected at the Wuhan Union Hospital by quantitative RT-PCR, ELISA, and immunohistochemical analysis (**Figures 12A–E**). The results were consistent with the database and mouse model results.

### Distribution of CD14, CD68, S100A8, and S100A9 in the aortic valve

**Figure 13:**
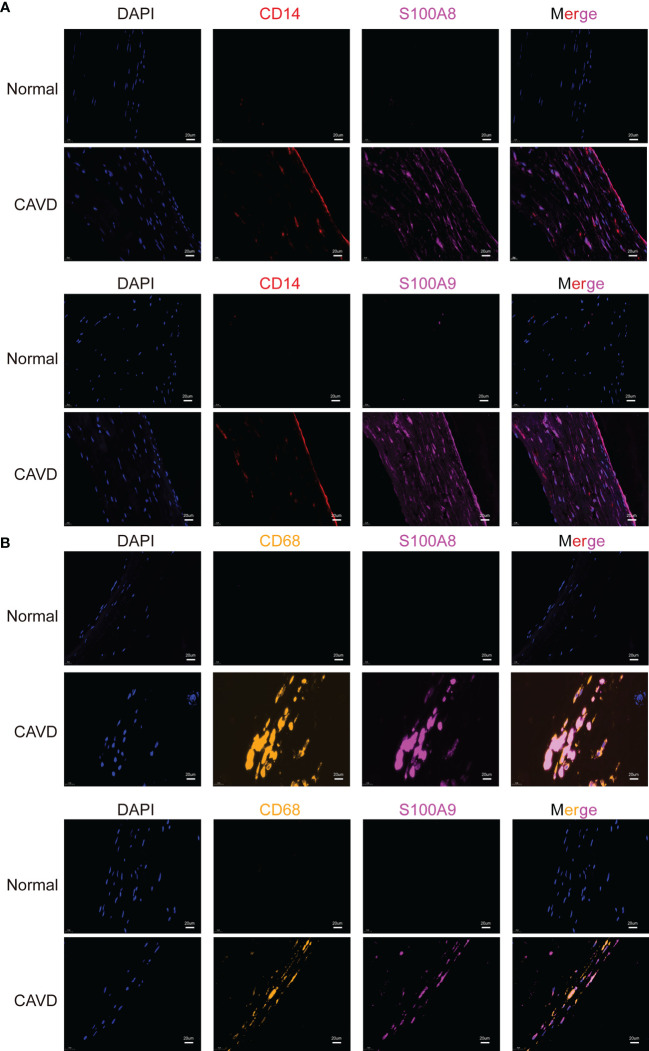
Colocalization of hub genes (*S100A8* and *S100A9*) and CD14 and CD68 staining. Immunofluorescence analysis of CD14/S100A8 and S100A9 **(A)**. The human aortic valve was stained with anti-CD14 (red) and anti-S100A8/S100A9 (purple) antibodies. S100A8 and S100A9 partly co-localized with monocytes. Immunofluorescence analysis showed CD68/S100A8 and S100A9 expression **(B)**. The human aortic valve was stained with anti-CD68 (yellow) and anti-S100A8/S100A9 (purple) antibodies. The scale bar represents 20 μm.

To confirm the cellular origin of S100A8 and S100A9, we performed hub gene (*S100A8* and *S100A9*) staining, and a monocyte biomarker (CD14) was used to locate monocytes in the human aortic valve (**Figure 13A**, **Supplementary File 2**). We found that CD14 partly co-localized with S100A8 and S100A9, which not only proved the S100A8 and S100A9 co-localization with monocytes but also suggested that they might co-localize with other cell types. This other cell type could be neutrophils (22, 23), which has also been confirmed in other studies in which S100A8/9 localization is consistent with the location of CD11b-positive cells (20, 24, 25), and CD11b serves as a marker for neutrophil activation. CD14 (a monocyte marker) is mainly expressed on the surface of the aortic valve, where it is in direct contact with the blood in the heart. These results were consistent with the scRNA-seq analysis results. In addition, a previous study showed that macrophages are associated with S100A8 and S100A9 in rats (21). Macrophages are phagocytic cells that differentiate from monocytes in tissues. Monocytes and macrophages are characterized by the expression of CD14. However, CD68 can be used to differentiate cell types, from monocytes to macrophages (26). Therefore, we performed an experiment to confirm the origin of S100A8 and S100A9. The results indicated that S100A8 and S100A9 co-localized with CD68 (a macrophage marker) (**Figure 13B**).

## Discussion

Owing to the large and aging population of China, a rapid increase in the incidence of CAVD has been observed in recent years (27, 28). To date, the only effective treatment that can improve CAVD symptoms is valve replacement (29). Elucidating the molecular mechanisms underlying calcific valves is urgently needed. With the marked advances in high-throughput technology, gene data have also increased tremendously in public databases. Hence, determining how to use data from different centers to help researchers determine the relationship between genes and diseases would represent an important direction for CAVD research (30).

With the aid of microarray and high-throughput sequencing technologies, previous studies have studied hub genes of CAVD and their functions in its development to provide new insights into this disease. However, these studies did not explain the molecular mechanisms responsible for the occurrence and development of CAVD (31–33). In this study, we not only analyzed the hub genes of CAVD based on a public database and single-cell sequence data from our center but also innovatively studied immune infiltration in CAVD.

In this study, we first integrated two CAVD datasets from the GEO database. In summary, 259 integrated DEGs were identified, including 191 upregulated and 68 downregulated genes. The results of the KEGG pathway analysis showed that DEGs associated with CAVD were enriched in “protein digestion and absorption”, “ECM–receptor interaction”, “AGE-RAGE signaling pathway in diabetic complications”, “focal adhesion”, and “cytokine–cytokine receptor interaction” terms. In the subsequent GO functional enrichment analysis, the results showed that the DEGs were primarily involved in “extracellular matrix organization”, “extracellular structure organization”, “external encapsulating structure organization”, “collagen catabolic process”, “extracellular matrix disassembly”, “neutrophil chemotaxis”, “collagen metabolic process”, “neutrophil migration”, “granulocyte chemotaxis”, and “antimicrobial humoral response” terms. We further identified two hub genes (*S100A8* and *S100A9*) that were significantly associated with CAVD by combining WGCNA module genes and DEGs in the public database.

To obtain insight into hub gene expression at the single-cell level and verify the transcriptomic results, six valve samples from our hospital were used for scRNA-seq (9). After quality control, the immune cells in the valve were divided into eight clusters, which could be approximately summarized as T cells, monocytes, B cells, NK cells, and platelets. We found that expression levels of hub genes (*S100A8* and *S100A9*) were significantly upregulated, especially in monocyte clusters.

Meanwhile, pseudotime analysis concluded that expression levels of these genes are upregulated in the final stage of immune cell differentiation. In addition, the final stage of immune cell differentiation in CAVD mainly comprises monocytes (**Figure 6C**), suggesting that S100A8 and S100A9 have a potential role in monocyte differentiation. Therefore, to assess monocyte differentiation, we extracted monocytes alone for single-cell analysis. Similarly, after monocyte cell data preprocessing, we found that these cells could be clustered into two subgroups, CD14+ monocytes and FCGR3A+ monocytes (**Supplementary File 3A–F**). We also found that S100A8 and S100A9 were highly expressed in CD14+ monocytes (**Supplementary File 3E, F**). Pseudotime analysis was then performed on monocyte cells, and the results indicated that S100A8 and S100A9 were mainly expressed in the early stage of monocyte differentiation. Finally, two hub genes were identified using scRNA-seq and GEO datasets. Notably, results based on three GEO datasets demonstrated that these two genes (*S100A8* and *S100A9*) might also act as accurate biomarkers for patients with CAVD based on the ROC (receiver-operating characteristic) curve. Finally, the localization of S100A8 and S100A9 was confirmed in monocytes and macrophages *via* immunofluorescence using human aortic valves.

Calgranulins (S100A8/9) and calprotectin (heterodimer of S100A8/9) belong to a family of 100 calcium-binding proteins (34). A S100A8/A9 heterodimer exists between S100A8 and S100A9 in the presence of Zn^2+^ and Ca^2+^. Yoon et al. reported that S100A8/9 could be used as a potential immunomodulatory agent (35). Studies have also revealed that S100A8/9 enhances inflammatory responses by mediating NF-κB activation and inducing cytokine secretion (36).

Hence, we explored the relationship between hub genes (*S100A8* and *S100A9*) and their possible mechanisms of action (immune checkpoints, m6A, cuproptosis, and ferroptosis). Only immune checkpoints were found to be most likely be associated with hub genes in CAVD, which is consistent with the results of previous studies (35, 36). Subsequently, with the aid of CIBERSORT, immune infiltration in CAVD was analyzed. We also found that M1 macrophage, Treg, plasma cell, and NK cell activation differed between the high and low hub gene expression groups. In the group with high hub gene expression, numbers of M1 macrophages, Tregs, plasma cells, and NK cells were higher, which provides a reference to further explore the immune microenvironment of CAVD. Previous studies have suggested that valvular calcification might be exacerbated in the context of inflammation (37), which is consistent with the increase in M1 macrophages in the high hub gene group.

Meanwhile, GSEA concluded that the high expression of hub genes was significantly associated with immune-related pathways, such as cell adhesion molecules, chemokine signaling pathway, and leukocyte transendothelial migration. Hence, these genes are potential candidate genes regulated by immune-related pathways involved in the pathogenesis of CAVD. Moreover, we verified the expression of hub genes (*S100A8* and *S100A9*) in clinical samples and ApoE−/− mice.

Indeed, much of the available data are available in the GEO database. The main reason that we chose newer datasets in this study is that sequencing technology has continued to improve in recent years, and newer data represent more reliable data. However, at the same time, owing to the exacerbation of sequencing data errors, if the dataset is continuously superimposed to obtain hub genes, the result will cause deviations and mask the actual results. The main purpose of our research was to conduct an external verification of our study. The three datasets from different sources support this result, which proves the accuracy of our conclusions.

In conclusion, using integrated bioinformatics analysis, such as WGCNA and scRNA-seq, we identified hub genes (*S100A8* and *S100A9*) as potential disease markers or therapeutic targets, which were not previously known to be associated with immune infiltration and CAVD. However, our experiment had some limitations. The functions of these genes in CAVD have not yet been validated based on molecular experiments, and we will address this in the future.

## Data availability statement

The datasets presented in this study can be found in online repositories. The names of the repository/repositories and accession number(s) can be found below: https://www.ncbi.nlm.nih.gov/, PRJNA562645.

## Ethics statement

The studies involving human participants were reviewed and approved by the Ethics Committee of Tongji Medical College, Huazhong University of Science and Technology. Written informed consent for participation was not required for this study in accordance with the national legislation and the institutional requirements. The animal study was reviewed and approved by the Animal Care and Utilization Committee of Tongji Medical College, Huazhong University of Science and Technology. Written informed consent was obtained from the individual(s) for the publication of any potentially identifiable images or data included in this article.

## Author contributions

KW conceived the research idea, study design, statistical analysis and writing of the manuscript. QZ performed animal experiments and assisted in the experiments. QZ, BG, and XL provided technical support and collected human tissues. BG provided comments on the structure of the article. ND and JS had read and provided final approval of the version to be submitted. The final manuscript was approved by all the authors above.

## Funding

This study was supported by the National Natural Science Foundation of China (grants 81770387) and the National Key Research and Development Plan of China (2021YFA1101900).

## Acknowledgments

We thank the Array Express, GEO, and GeneCards databases for the availability of the data.

## Conflict of interest

The authors declare that the research was conducted in the absence of any commercial or financial relationships that could be construed as a potential conflict of interest.

## Publisher’s note

All claims expressed in this article are solely those of the authors and do not necessarily represent those of their affiliated organizations, or those of the publisher, the editors and the reviewers. Any product that may be evaluated in this article, or claim that may be made by its manufacturer, is not guaranteed or endorsed by the publisher.

## Supplementary material

Supplementary File 1 Differentially expressed genes (DEGs) in the combined GEO datasets.

Supplementary File 2 Core genes of each cluster in single-cell analysis.

Supplementary File 3 Single-cell RNA sequencing (scRNA-seq) analysis of monocyte cells.

Supplementary File 4 Merged GEO data code (run with R 4.2.1. software).

Supplementary File 5 Differentially expressed genes (DEGs) in GSE153555 dataset.

Supplementary File 6 Hub genes in different modules.

Supplementary File 7 Summary of calcified aortic valve datasets from different GEO datasets.

Supplementary Figure 1 Expression of ferroptosis-related mRNAs in calcific aortic valve disease (CAVD) groups. **(A, B)** Comparison of expression levels of 22 ferroptosis-related RNAs. Ferroptosis-related RNA between S100A8/S100A9 high and low expression groups. Blue represents high expression, whereas a red represents low expression. **(C, D)** Visualization of differentially expressed ferroptosis-related mRNAs in CAVD. High S100A8/S100A9 expression in CAVD patients is marked blue, and low S100A8/S100A9 expression is marked red. **(E)** Spearman correlation analysis of 22 ferroptosis-related RNAs in CAVD. A higher number in the circle indicates a stronger correlation. The change in color on the right represents a positive or negative correlation. ^*^ *P* < 0.05, ^**^ *P* < 0.01, ^***^ *P* < 0.001 when two groups were compared as indicated or compared to the corresponding control.

Supplementary Figure 2 Expression of m6A RNA methylation regulators in calcific aortic valve disease (CAVD) groups. **(A, B)** Comparison of expression levels of 18 m6A RNA methylation regulators. m6A RNA methylation regulators between S100A8/S100A9 high and low expression groups. Blue represents high expression, whereas red represents low expression. **(C, D)** Visualization of differentially expressed m6A RNA methylation regulators in CAVD. High S100A8/S100A9 expression in CAVD patients is marked blue, and low S100A8/S100A9 expression is marked red. **(E)** Spearman correlation analysis of 18 m6A RNA methylation regulators in CAVD. A higher number in the circle indicates a stronger correlation. The change in color on the right represents a positive or negative correlation. ^*^ *P* < 0.05, ^**^ *P* < 0.01, ^***^ *P* < 0.001 when two groups were compared as indicated or compared to the corresponding control.

Supplementary Figure 3 Expression of cuprotosis-related mRNAs in calcific aortic valve disease (CAVD) groups. **(A, B)** Comparison of expression levels of 24 cuprotosis-related RNA. Cuprotosis-related mRNA expression between S100A8/S100A9 high and low expression groups. Blue represents high expression, whereas red represents low expression. **(C, D)** Visualization of differentially expressed cuprotosis-related mRNAs in CAVD. High S100A8/S100A9 expression in CAVD patients is marked blue, and low S100A8/S100A9 expression is marked red. **(E)** Spearman correlation analysis of 24 cuprotosis-related RNAs in CAVD. A higher number in the circle indicates a stronger correlation. The change in color on the right represents a positive or negative correlation. ^*^ *P* < 0.05, ^**^ *P* < 0.01, ^***^ *P* < 0.001 when two groups were compared as indicated or compared to the corresponding control.

## Abbreviations

CAVD, calcific aortic valve diseases; DEGs, differential genes; GEO, the Gene Expression Omnibus; GO, gene ontology; KEGG, Kyoto Encyclopedia of Genes and Genomes; S100A8/9, S100 calcium binding protein A8/9; scRNA-seq, single-cell sequence; WGCNA, weighted gene co-expression network analysis; qPCR, quantitative real-time polymerase chain reaction; WD, western diet; ND, normal diet.
