# Supplementary material for: Identifying hub genes of calcific aortic valve disease and revealing the immune infiltration landscape based on multiple WGCNA and single-cell sequence analysis
Source: Front Immunol. 2022 Nov 4;13:1035285. doi: 10.3389/fimmu.2022.1035285 (PMC9673246; doi:10.3389/fimmu.2022.1035285)
Supplement: Supplementary File 1 — Differentially expressed genes (DEGs) in the combined GEO datasets. [file DataSheet_1.zip › Supplementary figures and supplementary files 3, 4 and 7.DOCX]

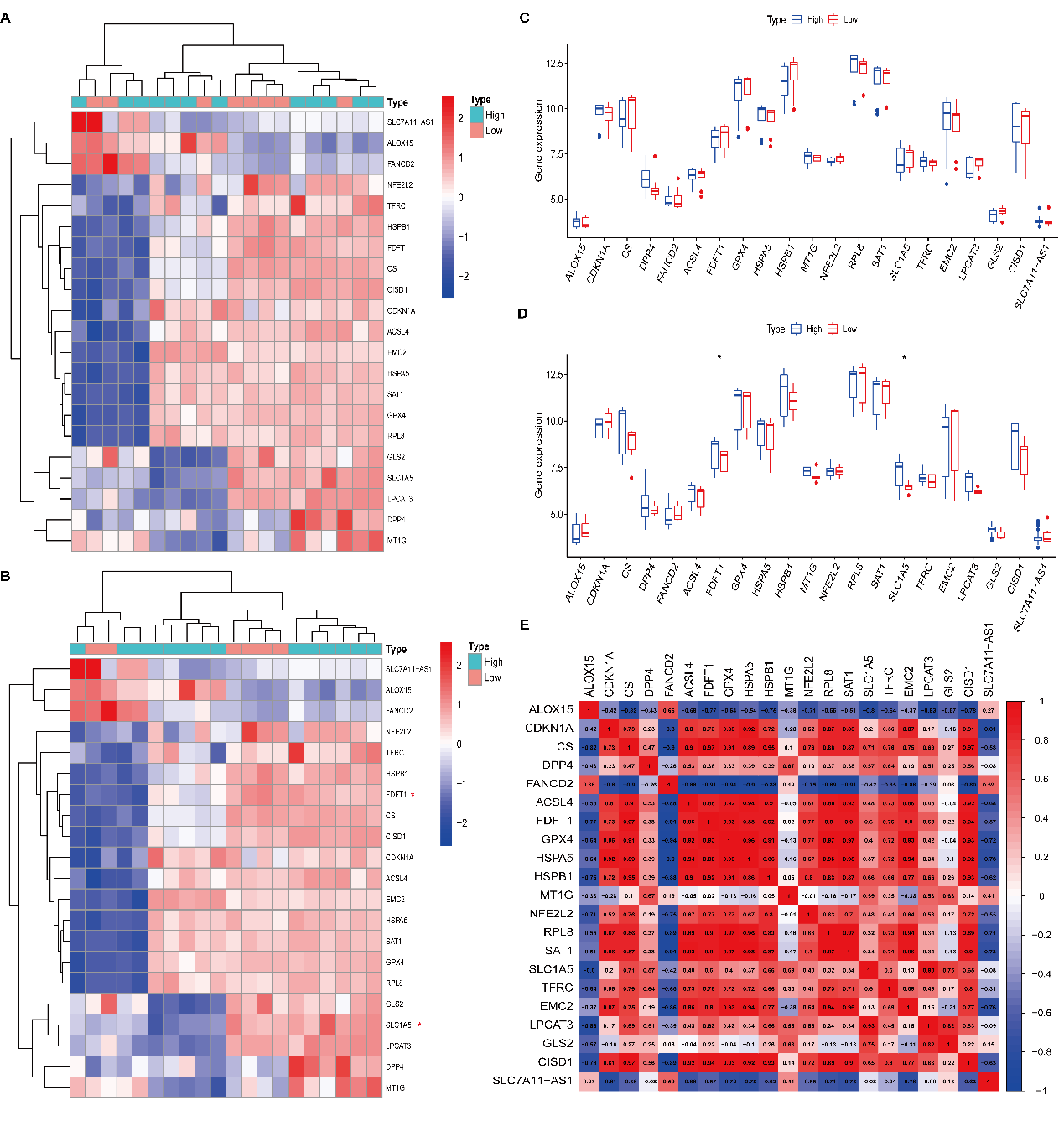


**Supplementary Figure 1. Expression of ferroptosis-related mRNAs in CAVD groups.**


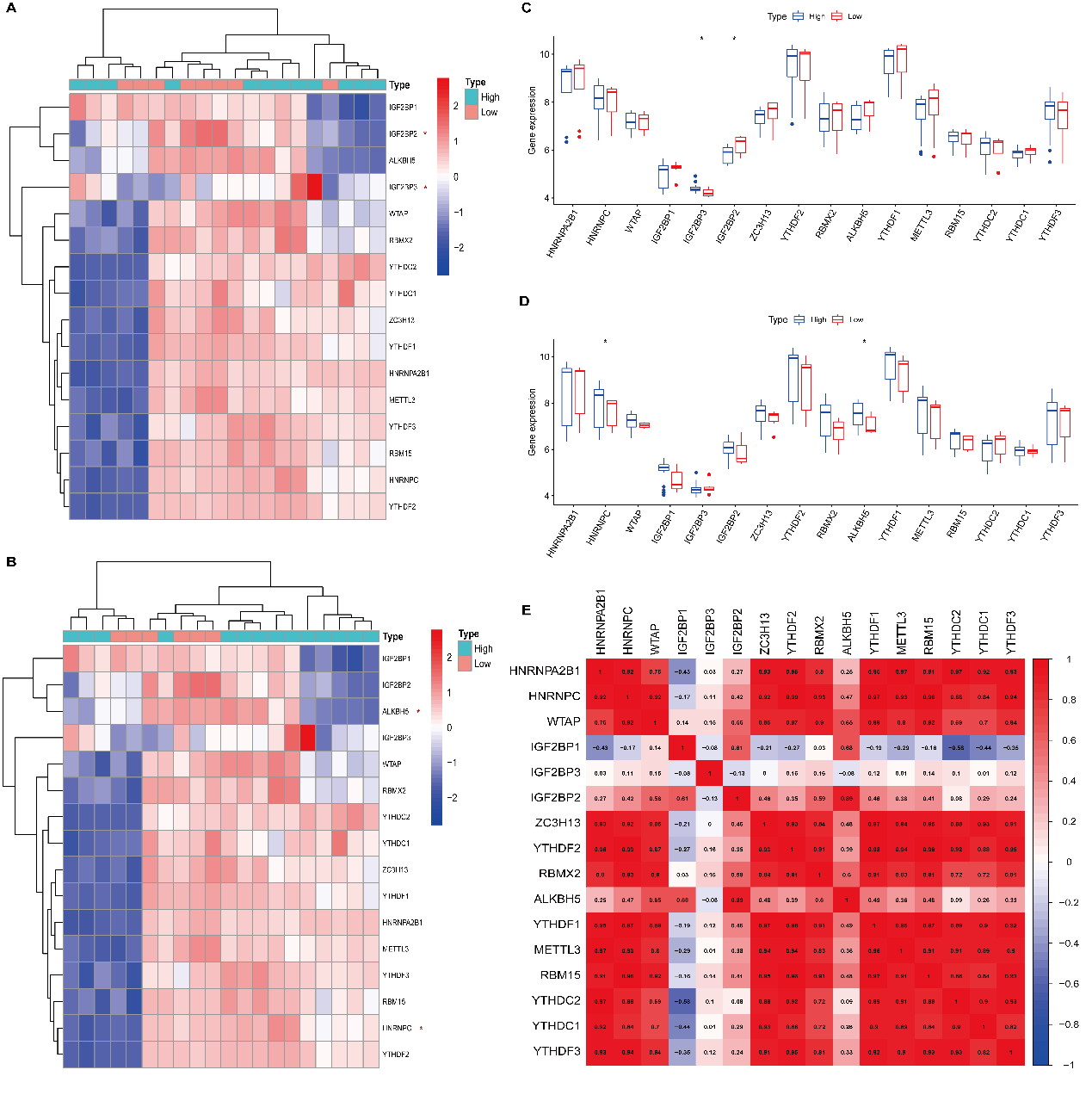


**Supplementary Figure 2. Expression of m6A RNA methylation regulators in CAVD groups.**


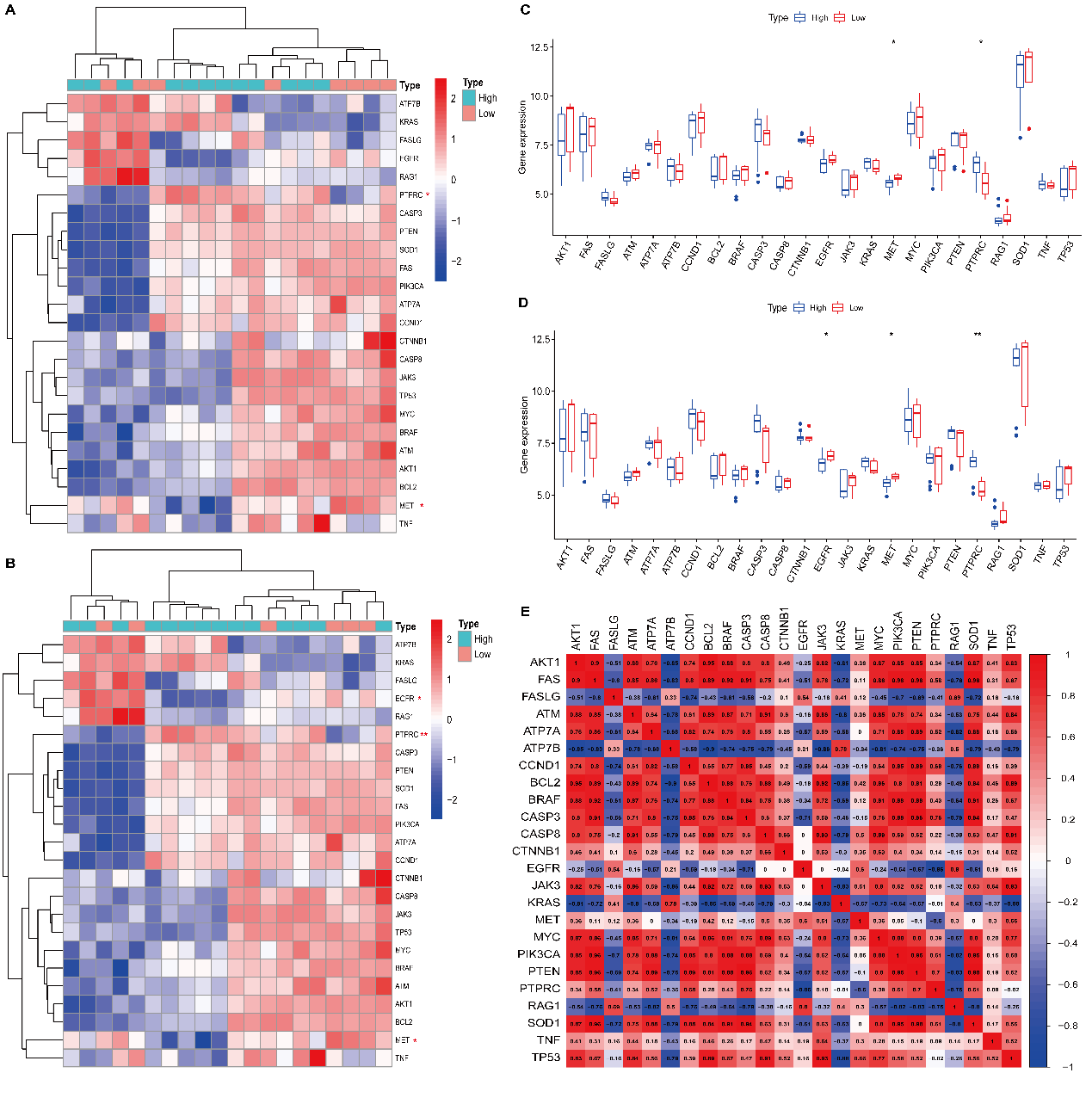


**Supplementary Figure 3. Expression of cuprotosis-related mRNAs in CAVD groups.**


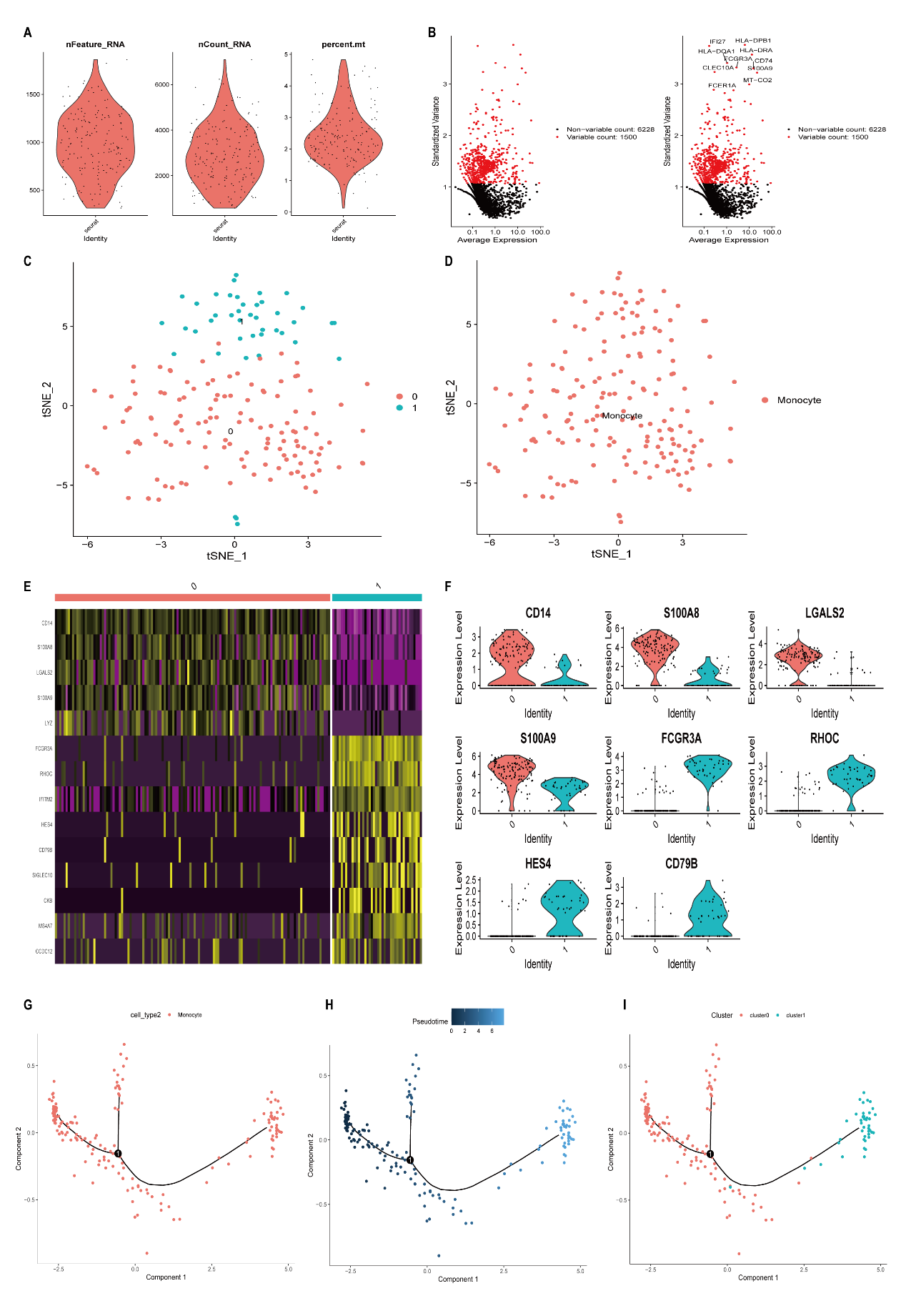


**Supplementary file 3. scRNA-seq analysis in monocyte cells.**

library(limma)

outFile="merge.txt"

files=dir()

files=grep("txt$", files, value=T)

geneList=list()

for(i in 1:length(files)){

inputFile=files[i]

if(inputFile==outFile){next}

rt=read.table(inputFile, header=T, sep="\t", check.names=F)

header=unlist(strsplit(inputFile, "\\.|\\-|\\_"))

geneList[[header[1]]]=as.vector(rt[,1])

}

interGenes=Reduce(intersect, geneList)

allTab=data.frame()

for(i in 1:length(files)){

inputFile=files[i]

if(inputFile==outFile){next}

header=unlist(strsplit(inputFile, "\\.|\\-|\_"))

rt=read.table(inputFile, header=T, sep="\t", check.names=F)

rt=as.matrix(rt)

rownames(rt)=rt[,1]

exp=rt[,1:ncol(rt)]

dimnames=list(rownames(exp),colnames(exp))

data=matrix(as.numeric(as.matrix(exp)),nrow=nrow(exp),dimnames=dimnames)

rt=avereps(data)

colnames(rt)=paste0(header[1], "_", colnames(rt))

if(ncol(allTab)==0){

allTab=rt[interGenes,]

}else{

allTab=cbind(allTab, rt[interGenes,])

}

}

outTab=rbind(geneNames=colnames(allTab), allTab)

write.table(outTab, file=outFile, sep="\t", quote=F, col.names=F)

**Supplementary file 4. The GEO data merged code** (run in R 4.2.1. software)**.**

| **Series** | **Platform** | **GeneChip** | **Normal** | **Calcification** | **Year** |
| --- | --- | --- | --- | --- | --- |
| GSE51472 | GPL570 platform | [HG-U133_Plus_2] Affymetrix Human Genome U133 Plus 2.0 Array | 5 | 5 | 2015 |
| GSE153555 | GPL16791 | Illumina HiSeq 2500 (Homo sapiens) | 10 | 20 | 2020 |
| GSE83453 | GPL10558 | Illumina HumanHT-12 V4.0 expression beadchip | 8 | 19 | 2016 |
| GSE12644 | GPL570 | [HG-U133_Plus_2] Affymetrix Human Genome U133 Plus 2.0 Array | 10 | 10 | 2009 |

**Supplementary file 7. A summary of calcified aortic valve datasets from different GEO datasets.**
